# Cutaneous leishmaniasis in the central provinces of Hama and Edlib in Syria: Vector identification and parasite typing

**DOI:** 10.1186/s13071-015-1147-0

**Published:** 2015-10-12

**Authors:** Nabil Haddad, Hanadi Saliba, Atef Altawil, Jeffrey Villinsky, Samar Al-Nahhas

**Affiliations:** Laboratory of Immunology, Faculty of Public Health, Lebanese University, Fanar, Lebanon; National Leishmaniasis Control Program, Ministry of Health, Damascus, Syria; US Naval Medical Research Unit No. 3, Cairo, Egypt; Department of Animal Biology, Faculty of Science, Damascus University, Damascus, Syria

**Keywords:** Cutaneous leishmaniasis, *Phlebotomus sergenti*, *Phlebotomus tobbi*, *Leishmania tropica*, *Leishmania infantum*, ITS1, RFLP, Edlib, Hama, Syria

## Abstract

**Background:**

Cutaneous leishmaniasis is a disease transmitted by sand fly bites. This disease is highly prevalent in Syria where *Leishmania major* and *Leishmania tropica* are the known aetiological agents. In 2011, more than 58,000 cases were reported in the country by the Ministry of Health. The central region of the country harbors 20 % of the reported cases. However, the epidemiology of the disease in this area is not well understood. An epidemiological survey was conducted in 2010 to identity the circulating parasite and the sand fly vector in the central provinces of Edlib and Hama.

**Methods:**

Sand fly specimens were collected using CDC light traps and identified morphologically. Total DNA was extracted from the abdomens of female specimens and from Giemsa-stained skin lesion smears of 80 patients. *Leishmania* parasites were first identified by sequencing the ITS1 gene amplicons. Then polymorphism analysis was performed using the RFLP technique.

**Results:**

A total of 2142 sand flies were collected. They belonged to eight species, among which *Phlebotomus sergenti* and *Phlebotomus papatasi* were the most predominant.

*L. tropica* ITS1 gene was amplified from two pools of *P. sergenti* specimens and from skin smears of cutaneous leishmaniasis patients. This suggests that *P. sergenti* is the potential vector species in the study area. The digestion profiles of the obtained amplicons by TaqI restriction enzyme were identical for all analysed *L. tropica* parasites. Moreover, *L. infantum* ITS1 gene was amplified from two pools of *Phlebotomus tobbi* in the relatively humid zone of Edlib.

**Conclusions:**

*L. tropica* is confirmed to be the aetiological agent of cutaneous leishmaniasis cases in the central provinces. RFLP technique failed to show any genetic heterogeneity in the ITS1 gene among the tested parasites. The molecular detection of this parasite in human skin smears and in *P. sergenti* supports the vector status of this species in the study area. The detection of *L. infantum* in *P. tobbi* specimens indicates a potential circulation of this parasite in the humid zone of Edlib. Further epidemiological studies are needed to evaluate the burden of this visceral parasite in the study region.

## Background

Cutaneous leishmaniasis (CL) is a disease caused by *Leishmania* parasites and transmitted to humans by sand fly bites in more than 70 countries around the world, in climates ranging from tropical to desert. Syria is considered one of the most endemic countries for CL [1]. Syrian health authority records show that the vast majority (90 %) of reported CL cases in the country are caused by *Leishmania tropica,* whereas the remaining cases are caused by the zoonotic parasite *L. major* [2]. Visceral leishmaniasis due to *Leishmania infantum* is also spread in Syria. The incidence for the period 2004–2008 was 14 cases/year, mainly reported from Edlib province. Dogs are suspected to be the main reservoir [2]. Published data from the Syrian Ministry of Health show that, despite the implementation of several control programmes, the incidence of CL has increased during the last fifteen years from 12,027 cases in 1997 to 58,156 cases in 2011 [3]. Since 2011, the ongoing Syrian war and the resulting massive population displacement have led to a significant increase of the incidence of CL in the country and in neighbouring Turkey, Jordan and Lebanon [4, 5]. In 2013, more than 1000 cases of CL (mainly caused by *L. tropica*) were recorded in Lebanon among Syrian refugees [4].

The northern province of Aleppo accounts for 56 % of CL, followed by the central provinces of Edlib and Hama which together harbor 20 % of all declared cases. Several studies on leishmaniasis have been conducted in the Aleppo area [6–10]. The causative agent was typed as *L. tropica* ZMON-76 [11]. However, the epidemiology of the disease in the central provinces remains poorly studied. Records from Hama province suggest that *L. tropica* is the main circulating parasite [12]. During the summer of 2010, we conducted an entomological survey in the provinces of Hama and Edlib in order to identify the potential vectors of CL and establish an inventory of existing sand fly species. In addition, we analysed Giemsa-stained smears of skin lesions belonging to CL patients residing in these provinces to determine the species identity and polymorphism of the circulating *Leishmania* parasites. Classically, polymerase chain reaction (PCR) based methods performed on various targets such as microsatellite, kinetoplast DNA, or ribosomal RNA have been used for identification of *Leishmania* isolates in various foci [13–15]. The internal transcribed spacers 1 (ITS1) gene, a ribosomal RNA gene, is considered a primary target for this purpose as shown in many studies conducted in endemic areas in the Old and New World [16–22]. In this study, sequence and restriction fragment length polymorphism (RFLP) analyses were performed on the ITS1 amplicons in order to identify the circulating species and to detect potential genetic polymorphism.

## Methods

### Description of the study area

**Fig. 1:**
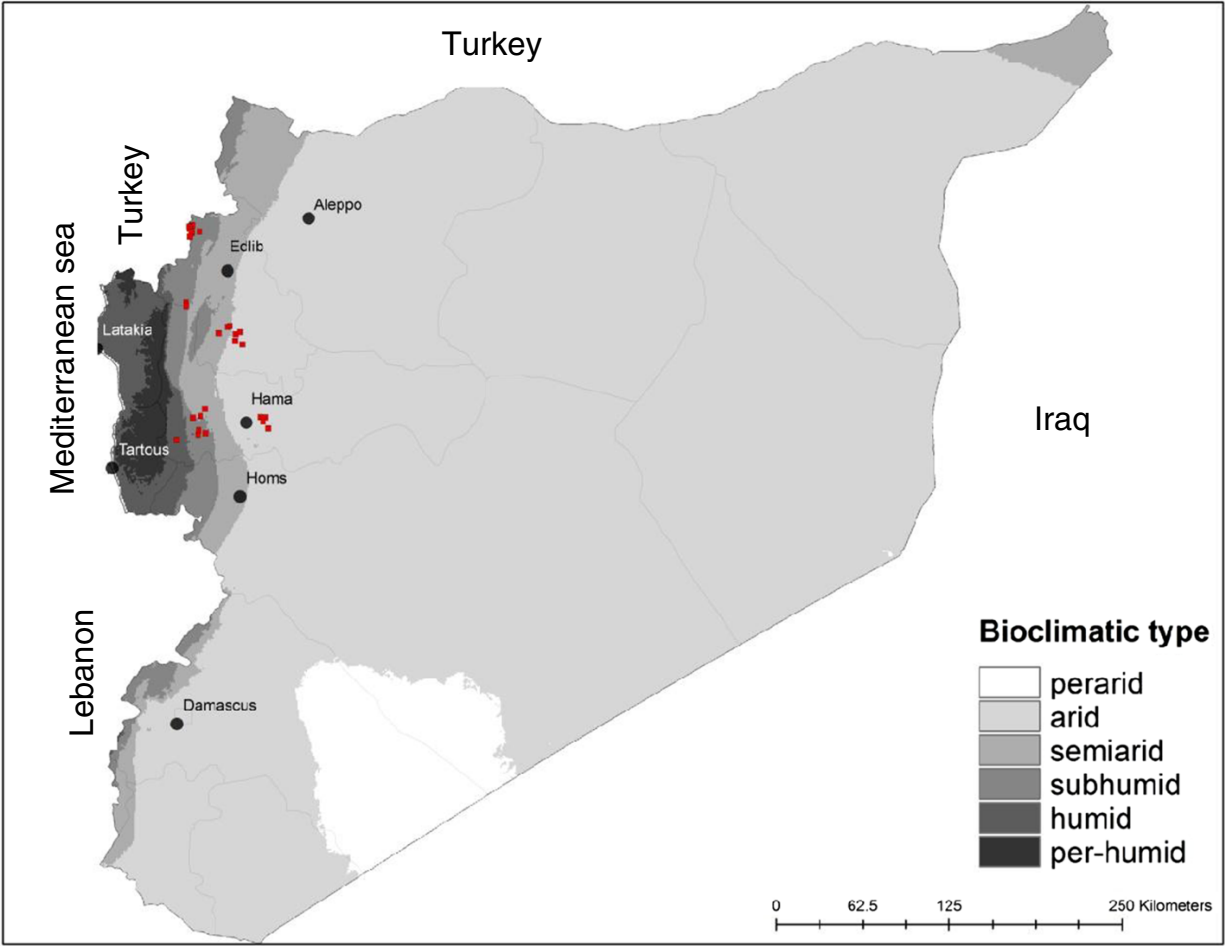
Sand fly collection sites (dots in red) in the provinces of Hama and Edlib

The provinces of Hama (8883 km^2^) and Edlib (5933 Km^2^) are located in the central region of Syria (Fig. 1), which is characterized by an arid climate with an annual average rate of precipitation lower than 200 mm. The western side of this region is influenced by the Mediterranean climate and, therefore, is obviously more humid. Agriculture is the main economic resource for the two million residents of this area.

### Sample collection

Sand flies were captured during July and August 2010, in 11 geographical locations in the province of Hama and 17 locations in the province of Edlib (Fig. 1), from where human CL cases were reported in 2010 by the Syrian Ministry of Health. CDC miniature light traps (John Hock Co., Gainesville, FL, USA) were placed in indoor (bedrooms, animal shelters) and outdoor habitats (private gardens). Collected specimens were preserved in 90 % ethanol. In addition, Giemsa-stained skin smear slides of patients with confirmed CL were provided to us by the Ministry of Health authorities. These smears were performed on skin lesions of patients seeking diagnosis and treatment at local primary health care centers in Hama and Edlib provinces. All tested smears were confirmed positive by observing *Leishmania* amastigotes microscopically.

### Sand fly identification

Male specimens were cleared in Marc-Andrée solution [23] and then mounted on slides in chloral gum medium [24]. Female specimens were dissected; their head and genitalia were cleared and then mounted, whereas their abdomen and thorax were preserved in ethanol for further molecular analysis. Female and male specimens were morphologically identified by observing head and genital structures under the microscope using local and regional identification keys [25, 26].

### DNA extraction

Abdomens of female sand flies were pooled (up to 10 per pool) according to species and capture locality. These samples were then ground in microtubes (1.5 mL). Slides of skin lesion smears were individually scraped using sterile blade scalpels. The scraping product was collected in microtubes. Total DNA was extracted from both types of materials using the extraction kit QIAamp DNA Mini Kit (Qiagen, Valencia, CA).

### PCR amplification and sequencing of ITS1 gene

DNA extracts were screened for *Leishmania* DNA by conventional PCR technique. A 339-bp fragment of the internal transcribed spacer region (ITS1) of the small subunit ribosomal DNA was amplified using specific primers: L5.8S: 5′-TGATACCACTTATCGCACTT-3′ and LITSR: 5′-CTGGATCATTTTCCGATG-3′ [17]. The PCR mix comprised 2.5 U Taq polymerase (Promega, Madison, WI), 0.6 pM of each primer, 1.5 mM MgCl_2_, 0.1 mM dNTPs, and 5 μL DNA template in a final volume of 50 μL. Cycling conditions were as follow: 95 °C, 120 s and (94 °C, 30 s; 53 °C, 60 s; 72 °C, 60 s) repeated 37 times.

Amplicons were visualized after electrophoresis in 1.5 % agarose gel in TBE (1X) buffer. DNA extracts from *L. infantum* parasite of the reference strain MHOM/FR/78/LEM 75 and male sand fly specimen were used respectively as positive and negative controls. Amplicons were purified using the Qiagen PCR Clean up Kit (Qiagen, Valencia, CA). Fifty fmol of purified DNA were sequenced in the forward direction with an *ABI Prism 3130 Genetic Analyzer*, Big Dye Terminator technology (Applied Biosystems, Foster City, CA, USA) using 1.6 pmol of each primer. Sequences were manually edited and used to query GenBank for species identity.

### RFLP analysis of amplified ITS1

In order to detect potential genetic polymorphism among the identified *Leishmania* parasites, 10 μL of non-purified ITS1 amplicons were digested with Taq I restriction enzyme as indicated by the manufacturer (New England Biolabs, Ipswich, MA, USA). The digested products were run on 1.5 % electrophoresis gel agar at 100 V in TBE buffer and visualized under ultraviolet light. Bands were stained by adding GelRed dye (Phenix research, USA) to the gel preparation.

## Results

### Identification of collected sand fly specimens

The total number of sand flies captured was 2142, including 603 in Hama province and 1539 in Edlib province. Their identification revealed eight species, of which seven belonged to the *Phlebotomus* genus and one to the *Sergentomyia* genus (Tables 1 and 2).

**Table 1:** Species identity of sand fly specimens collected from 11 localities in the province of Hama

Collection locality	Geographical coordinates		*P. (Ph) papatasi*	*P. (Para) sergenti*	*P. (Para) jacusieli*	*P. (L) tobbi*	*P. (L) gallilaeus*	*P. (L) syriacus*	*S. (S) dentata*	*Total*
Aouja	35° 9'13.26"N	36°52'52.99"E	15	5	0	0	0	0	20	40
Asileh	35°11'57.51"N	36°29'16.17"E	4	10	0	0	1	0	5	20
Deir El Salib	35° 5'9.76"N	36°26'49.24"E	8	41	0	5	0	3	2	59
EL Rasafah	35° 1'58.98"N	36°18'8.62"E	6	24	4	4	0	4	9	51
Hamadi El Chihan	35° 7'54.30"N	36°51'55.38"E	3	1	0	0	0	0	0	4
Kaskas	35° 4'7.30"N	36°29'28.72"E	28	231	0	2	0	3	2	266
Kasoun El Jabal	35° 9'17.65"N	36°50'50.02"E	7	4	0	0	0	0	2	13
Kfarakid	35° 3'44.01"N	36°26'34.99"E	11	31	1	9	0	2	1	55
Maarrine	35° 9'40.38"N	36°27'24.21"E	7	11	0	0	1	0	3	22
Mahrouseh	35° 9'9.60"N	36°24'34.05"E	8	31	0	4	0	0	3	46
Smakh	35° 5'39.27"N	36°53'56.72"E	9	16	0	0	0	0	2	27
Total			106	405	5	24	2	12	49	603

Letters between brackets indicate the species subgenus: *(Ph) Phlebotomus*, *(Para) Paraphlebotomus*, *(L) Larroussius*, *(S) Sergentomyia*

**Table 2:** Species identity of sand fly specimens collected from 17 localities in the province of Edlib

Collection locality	Geographic coordinates		*P. (Ph) papatasi*	*P. (Para) sergenti*	*P. (Para) jacusieli*	*P. (Para) alexandri*	*P. (L) tobbi*	*P. (L) gallilaeus*	*P. (L) syriacus*	*S. (S) dentata*	Total
Al Allani	36° 9'39.87"N	36°23'7.62"E	0	0	0	0	6	0	0	0	6
Al Bakdash	35°44'38.41"N	36°21'53.93"E	12	13	0	0	7	1	1	17	51
Biskla	35°36'0.23"N	36°34'47.02"E	12	19	0	0	2	0	1	0	34
Deir Charki	35°36'18.17"N	36°42'53.65"E	3	18	0	0	0	0	0	0	21
Deir Gharbi	35°35'34.94"N	36°41'12.83"E	28	129	0	0	0	0	0	7	164
Delbieh	36° 7'38.69"N	36°24'8.31"E	7	0	0	0	4	0	0	0	11
El Jakarah	36° 8'55.49"N	36°23'20.67"E	7	0	0	0	2	1	0	1	11
El Jebb	36°10'7.98"N	36°24'16.58"E	16	98	0	0	9	4	5	0	132
El Kah	35°32'25.94"N	36°43'44.11"E	17	44	0	0	1	0	0	23	85
El khamrieh	36° 6'30.90"N	36°23'21.13"E	29	2	0	1	31	270	12	0	345
El Safsafeh	36° 8'9.89"N	36°24'0.53"E	3	0	0	1	23	11	4	1	43
Freikheh	35°45'40.77"N	36°21'48.07"E	2	2	0	0	0	0	0	0	4
Hass	35°37'59.47"N	36°38'1.72"E	42	13	1	0	1	0	2	2	61
Kafroumeh	35°38'15.85"N	36°39'1.24"E	138	248	0	0	2	0	4	23	415
Sallet El Zouhour	35°44'53.77"N	36°21'48.74"E	5	4	0	0	3	0	0	2	14
Tekaneh	35°33'31.92"N	36°41'3.85"E	31	66	0	0	1	0	0	0	98
Wadi el Dahab	36° 8'6.74"N	36°27'10.03"E	1	0	0	0	10	23	0	10	44
Total			353	656	1	2	102	310	29	86	1539

Letters between brackets indicate the species subgenus: *(Ph) Phlebotomus*, *(Para) Paraphlebotomus*, *(L) Larroussius*, *(S) Sergentomyia*

The dominant species among collected specimens was *Phlebotomus sergenti* in both provinces, followed by *Phlebotomus papatasi*. These species are known vectors of anthroponotic and zoonotic forms of CL respectively [27–32]. *Larroussius* subgenus is dominantly represented by *Phlebotomus gallilaeus*. Specimens of this species were mainly collected from the locality of Khamrieh in the humid area west of Edlib, near the Turkish border. *Phlebotomus tobbi* is another *Larroussius* member found in the study area. This species is spread mainly in countries of the mid-northern to eastern sides of the Mediterranean basin. Moreover, *P. tobbi* is the vector of *L. infantum* in Cyprus [33] and a suspected vector of this parasite in several countries, including Turkey and Iran [34, 35].

### Parasite identification

**Fig. 2:**
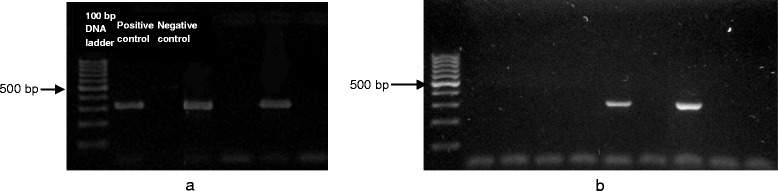
Electrophoresis gel showing PCR products of the ITS1 gene amplified from (**a**) two sand fly pools of *P. sergenti* (Lanes 4 and 6) (**b**) two sand fly pools of *P. tobbi* (Lanes 6 and 8)

The presence of ITS1 gene of *Leishmania* parasite was investigated by PCR on 1172 specimens of female sand flies, divided into 216 pools, as well as on skin smears of 80 patients of CL (40 from each province). Amplification results identified two positive pools of *P. sergenti,* one collected in the locality of Deir Charki (Edlib), the other in Smakh (Hama) (Fig. 2). In total, 489 female specimens of this species were tested in the study zone, including 254 from Edlib province (of which 14 were from Deir Charki) and 235 from Hama province (16 from Smakh). We also identified two *Leishmania* positive pools of *P. tobbi*, which were collected in the localities of El Jebb and El Safsafeh in Edlib. In this province, a total of 85 female specimens of *P.tobbi* were collected, of which only 6 were captured in El Jebb and 19 in El Safsafeh. On the other hand, ITS1 gene amplicons were obtained from 70 skin smears (34 from Edlib and 36 from Hama) out of the 80 tested samples.

All the obtained amplicons were sequenced. Their analysis revealed that the gene segments amplified from the two pools of *P. sergenti* and those from all analysed skin smears from both Syrian provinces are identical and belong to *L. tropica.* These sequences perfectly matched with ITS1 sequences of *L. tropica* isolate MHOM/AF/88/KK27 from Afghanistan and MHOM/IR/2012/Savodjbolagh isolate from Iran [GenBank: GQ913688.1, GenBank: KC574386.1]. Moreover, analysis of ITS1 gene sequences amplified from the two pools of *P. tobbi* from Edlib showed that the parasite belongs to *L. infantum* and is identical to the Iranian strain ITOB/IR/2009/Bilesavar [GenBank: HQ535858.1], which was also isolated from *P. tobbi*.

### RFLP results

**Fig. 3:**
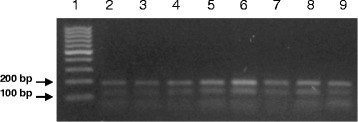
Gel electrophoresis of RFLP analysis. Lane 1: 100 bp DNA ladder; Lanes 2–9: Digestion profile by Taq1 α enzyme of the ITS1 gene fragments amplified from smears of skin lesions

After species identification, all PCR products of *L. tropica* from the skin smears of CL patients and from *P. sergenti* pools were digested by Taq1 restriction enzyme in order to detect potential genetic polymorphism. Digestion products showed that all isolates displayed two DNA bands of approximately 140 and 190 bp (Fig. 3).

## Discussion

The objectives of the current study were to identify the sand fly vectors and *Leishmania* species responsible for the CL cases in the central provinces of Hama and Edlib. Sequences of the ITS1 gene amplified from skin lesions of 70 patients showed that *L. tropica* is the causative agent of CL in both provinces. This species is also the main agent of CL in the highly endemic Aleppo province, northern Syria [9, 36] and in several foci in neighbouring countries [37, 38]. PCR-RFLP analysis of the ITS1 gene is commonly used to detect intraspecific genetic diversity among *L. tropica* species [21, 22]. This method is usually used as an alternative to sequence comparison by alignment. RFLP results revealed that all analysed strains from the study area had identical digestion patterns of the ITS1-gene, characterized by two DNA bands. This suggests the absence of mutations on the restriction site of the Taq1 enzyme. The absence of other polymorphic sites on the ITS1-gene was confirmed by sequence alignment (data not shown). Nevertheless, the existence of genetic diversity on other gene markers, such as Kinetoplast DNA minicircle, remains a possibility and requires further investigation. Our findings corroborate the observation of Pratlong and collaborators (2009) who identified only one *L. tropica* zymodeme (MON-76) among 51 tested samples [39]. Whereas, in Iran, Doudi and collaborators (2012) [22] were able to detect one dominant *L. tropica* isolate characterized by a specific digestion pattern using Taq 1 enzyme, in addition to two other types of isolates with different digestion patterns, reflecting the existence of a genetic polymorphism at the level of the ITS1 gene. Moreover, our results suggest that the sand fly vector responsible for *L. tropica* transmission in the study area is *P. sergenti*. Two pools of sand flies belonging to this species were positive for *Leishmania* ITS1 gene. PCR-RFLP analysis of the amplified gene produced the same digestion pattern as that observed for all *L. tropica* isolates from skin lesions. Besides, *P. sergenti* is the dominant sand fly (43 %) in our collections. This species is known to be the vector of *L. tropica* in several countries [27–30]. *L. tropica* is an anthroponotic parasite; nevertheless, zoonotic transmission has been documented in certain foci in the Middle East. The rock hyrax (*Procavia capensis*) is the main reservoir of *L. tropica* in certain Israeli and Palestinian foci [40]. In addition, dogs (and other Canidae) are suspected to be involved in the transmission of this *Leishmania* species in Morocco, Iran and Israel [41–43]. The high endemicity of *L. tropica* in the central provinces in Syria suggests an anthroponotic transmission pattern of this parasite. However, the involvement of dogs as reservoirs should be investigated. It is worth noting the absence of *L. major*, the agent of zoonotic CL, among the tested skin smears, despite the important spread of its specific vector, *P. papatasi*. The rodent reservoir of this species (*Psammomys obesus*) does not occur in the central provinces, while it is found mainly in the desert area, east of Damascus [44].

*L. infantum* seems to circulate in the study area, as it was detected in two pools of female specimens of *P. tobbi* collected in the humid area of Edlib. *L. infantum* is the known agent of visceral leishmaniasis in the Mediterranean basin. Few cases of VL are reported annually in Syria. Recent reports showed an incidence of VL of 14 cases per year, based on a 2–4-fold underreporting ratio [1]. The involvement of *P. tobbi* in the transmission of visceral *Leishmania* parasites was also recorded during a study conducted in the province of Lattakia that borders the western side of the study zone [45]. Nevertheless, *L. infantum* was also proven to be a causative agent of CL in the Mediterranean basin [46–50]. In the neighbouring Anatolia (South of Turkey), Svobodova and collaborators (2009) identified *L. infantum* as the causative agent of cutaneous lesions [33]. These authors incriminated *P. tobbi* as the vector of this dermotropic form. We expect a similar epidemiological situation in the humid zones west of Syria. Analysis of skin smears of patients originating from these zones is required to confirm the circulation of dermotropic *L. infantum*.

## Conclusions

Using PCR-ITS1 method*, L. tropica* was identified as the parasitic agent responsible for the high number of CL cases in the central provinces of Syria. RFLP analysis of the ITS1 gene amplicons obtained from skin lesions and from the sand fly vector did not reveal any polymorphism among the circulating parasites. Entomological data showed that *P. sergenti* and *P. papatasi* are the most dominant species in our collections. The detection, for the first time in Syria, of *L. tropica* ITS1 gene in two pools of *P. sergenti,* supports the vector status of this species. The amplification of *L. infantum* ITS1 gene from two pools of *P. tobbi* in the humid part of Edlib suggests an active circulation of this parasite. Further studies in humans, vectors and potential reservoirs are needed to determine the burden of *L. infantum* in the study area and in other parts of Syria.
